# Novel Agents For Relapsed and Refractory Classical Hodgkin Lymphoma: A Review

**DOI:** 10.3389/fonc.2022.929012

**Published:** 2022-07-14

**Authors:** Yujie Zhang, Zhichao Xing, Li Mi, Zhihui Li, Jingqiang Zhu, Tao Wei, Wenshuang Wu

**Affiliations:** ^1^ Department of Thyroid Surgery, West China Hospital, Sichuan University, Chengdu, China; ^2^ Laboratory of Thyroid and Parathyroid Disease, Frontiers Science Center for Disease-Related Molecular Network, West China Hospital, Sichuan University, Chengdu, China

**Keywords:** novel agents, brentuximab vedotin, nivolumab, pembrolizumab, relapsed and refractory classical hodgkin lymphoma

## Abstract

Classical Hodgkin lymphoma (cHL) is the most common type of HL that occurs mainly in people aged between 15–30 and over 55 years. Although its general prognosis is favorable, 10%–30% of patients with cHL will ultimately develop relapsed or refractory disease (r/r cHL). Improving the cure rate of r/r cHL has proven to be challenging. Some novel agents, such as brentuximab vedotin and immune checkpoint inhibitors, which have been used in conventional regimens for patients with r/r cHL in the past decade, have been shown to have good curative effects. This paper reviews the conventional regimens for patients with r/r cHL and focuses on the newest clinical trials and treatment measures to prolong prognosis and reduce adverse events. The evaluation of prognosis plays a vital role in analyzing the risk of relapse or disease progression; thus, finding new predictive strategies may help treat patients with r/r cHL more efficaciously.

## 1 Introduction

Hodgkin lymphoma (HL) is a frequent hematological malignancy, with 8,830 new cases reported in the United States in 2021 (1). Classical HL (cHL) is one of the most common types of HL and is defined as a B lymphatic cell malignancy characterized by the presence of malignant Hodgkin and Reed–Sternberg (HRS) cells in the tumor microenvironment (2). It occurs mainly in people aged between 15–30 and over 55 years, with an incidence of approximately three newly diagnosed cases per 100,000 individuals per year (3, 4). cHL is mainly divided into stages I–II and stages III–IV, the former of which is further classified into three subgroups, namely, stages IA–IIA (favorable), stages I–II (unfavorable with non-bulky disease), and stages I–II (unfavorable with bulky disease) (5). Patients with different stages may have different therapeutic choices. However, the prognosis of patients with HL is generally favorable, with approximately 80% of young adults cured of the disease after receiving initial standard chemotherapeutic treatment (3). However, 10%–30% of patients ultimately develop relapsed or refractory cHL (r/r cHL) disease (3). Patients with r/r cHL, especially those aged 60 years or older, have a much poorer prognosis, with a reported 3-year progression-free survival (PFS) rate of 50% and an overall survival (OS) rate of 68% (6). In addition, the toxic effects of the therapeutic approach on the number of years lost from productive life are remarkable. The common adverse events of conventional chemotherapeutic agents, such as peripheral neuropathy (7), also negatively affect the prognosis. Therefore, finding novel agents and therapeutic modalities is still of great significance in improving the overall cure rates and prolonging PFS and OS in patients with r/r cHL. Herein, we introduce the current chemotherapies used in clinical practice and novel treatments that show improved prognosis in the latest clinical trials to provide a more comprehensive understanding of r/r cHL treatment and promising directions for future research in this field.

## 2 Current standard treatment of r/r cHL

### 2.1 Treatment for adult patients aged 18–60 years

Combined modality therapy (chemotherapy and radiotherapy) remains a top priority for patients with newly diagnosed cHL. For stage I–II patients aged 18–60, two cycles of ABVD (doxorubicin, bleomycin, vinblastine, and dacarbazine) followed by positron emission tomography/computed tomography (PET/CT) evaluation are routinely used as the primary treatment. Further decisions regarding combined therapy are based on lymphoma remission status assessed by PET/CT. The escalated BEACOPP (bleomycin, etoposide, doxorubicin, cyclophosphamide, vincristine, procarbazine, and prednisone in escalated doses) regimen is also widely used in early-stage young adults as an additional therapy. For stage III–IV patients, apart from the ABVD regimen, escalated BEACOPP or brentuximab vedotin (BV) combined with AVD (doxorubicin, vinblastine, and dacarbazine) is recommended as the primary treatment in certain cases (8). However, the prognosis is significantly poorer for a small proportion of patients who may have a limited response to first-line therapy or relapse. Therefore, salvage treatment, high-dose therapy, and autologous stem cell transplantation (HDT-ASCT) play a key role in prolonging PFS and OS.

Patients with r/r cHL are recommended to be treated with salvage regimens, which mainly include dexamethasone, cisplatin, high-dose cytarabine (DHAP); etoposide, methylprednisolone, high-dose cytarabine, cisplatin (ESHAP); ifosfamide, carboplatin, etoposide (ICE); gemcitabine, vinorelbine, liposomal doxorubicin (GVD); BV; and a combination of bendamustine and nivolumab (5). A brief summary of the newly reported clinical trials on salvage systemic therapies is listed in **Table 1**. If PET/CT re-evaluation indicates a complete response (CR) or partial response (PR), additional therapy with HDT-ASCT would be feasible and effective if not contraindicated. Radiation should also be considered for selected sites that have not previously been irradiated. Response to salvage treatment is an important predictor of prognosis in patients with r/r cHL since individuals achieving metabolic CR after salvage treatment have a higher chance of being cured (9). In contrast, patients who experience disease progression after salvage treatment may benefit from further systemic therapy with or without radiation, and if they respond, autologous or allogeneic hematopoietic stem cell transplantation (HSCT) is also favorable. However, half of the patients who undergo HSCT cannot be cured, and their prognosis is poor (10). Therefore, maintenance therapy, such as BV and other novel modalities, is vital for patients at a high risk of relapse. For those who underwent multiple-line therapy and still suffered from disease relapse, regimens such as chimeric antigen receptor (CAR) T-cell therapy (11) may offer sustained relief.

**Table 1:** Newly reported clinical trials of salvage treatment of r/r cHL.

Agents	Patients	No. of patients	CR (%)	ORR (%)	Severe toxicity rate (%)	NCT number
V	Relapsed or ineligible post-ASCT	153	24	54.2	11	NCT02684292
BV + bendamustine	Refractory or relapsed after multiple-line chemotherapy	37	43	78	14 (lung infections) 25 (neutropenia)	NCT01657331
BV + nivolumab	Refractory or relapsed after first-line chemotherapy	93	67	85	18 (immune-related)	NCT02572167
BV + ESHAP	Refractory or relapsed after first-line chemotherapy	66	70	91	33	–
BV + ICE	Refractory or relapsed after first-line chemotherapy	45	74	91	29	NCT02227199
Pembrolizumab	Relapsed or ineligible post-ASCT	210	27.6	71.9	12.0	NCT02453594
Pembrolizumab	Relapsed or ineligible post-ASCT	151	25	65.6	16	NCT02684292
Pembrolizumab + GVD	Refractory or relapsed after first-line chemotherapy	39	95	100	31	NCT03618550

### 2.2 Treatment for older patients

cHL in patients over 60 years of age is highly linked to poorer prognosis since these patients generally suffer from inferior efficacy but great toxicity, owing to comorbidity and geriatric fitness (12). The management of older patients should be individualized and usually requires clinical judgment. Indeed, choosing agents with mild toxicities and at suitable doses remains challenging when aiming to improve the OS and PFS of this group of patients (5). ACCRU, as a multicenter phase II trial, reported the combination of BV and nivolumab as first-line therapy in older patients, and 22 out of 46 (48%) individuals achieved complete response, although grade 3–5 adverse events occurred in 80% of the patients (13). However, for older patients with r/r cHL, no consensus has been reached regarding the suggested treatment regimens.

### 2.3 Treatment for pediatric patients

Regimen options for pediatric cHL patients are distinct from those for adult patients, and the treatment of pediatric patients is highly based on risk stratification (14, 15). OEPA (vincristine, etoposide, prednisone, and doxorubicin), AVPC (doxorubicin, vincristine, prednisone, and cyclophosphamide), and ABVE-PC (doxorubicin, bleomycin, vincristine, etoposide, cyclophosphamide, and prednisone) regimens are conventionally used in underage patients as first-line systemic therapy (16) but are rarely used in adult patients. In contrast, for adolescents with suspected r/r cHL, available clinical trials are favored if the biopsy site is positive. Otherwise, re-induction therapy is required to avoid relapse or prolong PFS. HDT-ASCT, radiotherapy, and maintenance therapy are optional based on the metabolic condition of patients. Subsequent therapy was considered when relapse occurred after maintenance treatment. Re-induction and subsequent therapy are similar to those in adults, such as DHAP, IGEV (ifosfamide, gemcitabine, vinorelbine), ICE, nivolumab (17), BV (18), and the combination of BV with bendamustine, gemcitabine, or nivolumab.

## 3 Latest Updates of Targeted Agents for r/r cHL

### 3.1 BV

BV is a CD30-directed antibody–drug conjugate (ADC) that links an antineoplastic agent, monomethyl auristatin E (MMAE), to a monoclonal antibody that can direct MMAE to CD30-positive lymphoma cells (19). BV was initially approved for patients who failed ASCT or at least two prior lines of chemotherapy by the US Food and Drug Administration (FDA) in 2011, based on its great drug efficacy in a phase II clinical trial, with an overall response rate (ORR) of 75% (20). BV is now commonly used as salvage or post-ASCT maintenance therapy for patients with r/r cHL, but it has also been proven to be effective and recommended in patients with stage III/IV cHL as first-line therapy when combined with the AVD modality (8). Notably, severe adverse events of BV are negligible due to a high incidence rate of 32%, among which the most common and severe one is neutropenia (21). The latest update of the ECHELON-1 study illustrated that the BV plus AVD regimen could significantly improve the 5-year PFS compared to the standard ABVD regimen for the first-line treatment of patients with stage III/IV cHL (82.2% vs. 75.3%, *p* = 0.0017) (22). Another phase II trial further investigated the outcomes of BV-AVD treatment in patients with non-bulky stage I/II cHL, concluding a favorable PFS (94%) and OS (97%) during a median follow-up of 38 months (23).

For salvage treatment, BV monotherapy administered prior to ASCT in the treatment of r/r cHL yielded a CR rate (CRR) of 24%–35% (24, 25), which is not a favorable outcome, probably due to BV chemoresistance (26). In contrast, BV combined therapy has been intensively studied and demonstrated to markedly improve CR (25, 27, 28). Clinical trials of BV combined with conventional salvage chemotherapy have been widely performed over the last decade, including the combination of BV with ICE, DHAP, and ESHAP. The concurrent treatment with BV and ICE produced a high CRR of 74% and improved post-HSCT outcomes (29). Bendamustine is a purine analog with antitumor activity by damaging DNA and inducing cell apoptosis and has been approved for the treatment of chronic lymphocytic leukemia and non-HL. The combination of BV and bendamustine (BVB) as salvage therapy has shown a high CRR of 73.6% (30). BVB therapy has been widely researched in developed countries and has displayed impressive outcomes as a second-line treatment, with a CR of over 70% (30). In comparison, the CR of BVB combined therapy in a middle-income setting in India was lower at 62% (31). BVB is also highly active in patients with prior BV exposure since the PFS duration was similar to that of patients that had not received BV before (32). Another retrospective analysis indicated that BVB salvage therapy is highly effective in children and young adults under 30, with a high CR of 79% (33). A phase II transplant BRaVE trial recruited 55 patients with r/r cHL and administered BV-DHAP combined therapy as the first salvage treatment. CR was achieved in 81% of the patients before HDT-ASCT (34), which is a high rate compared to BV in combination with bendamustine (73.6%) (30) and ICE (74%) (29).

For maintenance therapy, the phase 3 AETHERA trial demonstrated the efficacy of BV in patients with a high risk of relapse after auto-HSCT treatment. The 5-year PFS was 59% in BV-treated patients compared with 41% in the placebo group [hazard ratio (HR), 0.521; 95% CI, 0.379–0.717] (35). Maintenance therapy with BV is comparatively safe, and 90% of the patients are diagnosed with peripheral neuropathy, which is the most common adverse event of BV (35, 36). The AETHERA trial showed that BV is an effective post-ASCT maintenance agent. However, since the AETHERA trial excluded individuals who had previously received BV, the efficacy of BV in this group remains unclear. A recent multicenter retrospective study of 105 cases, including both BV-naive and BV-exposed patients, reported 3‐year PFS and OS rates of 54% and 71%, respectively, for BV-naive patients and 77% and 96%, respectively, for BV-exposed patients (37).

### 3.2 Immune checkpoint inhibitors

Immune checkpoint inhibitors (ICIs) have been widely used to treat hematologic malignancies, including cHL. Anti-PD-1 agents have been widely used to treat cHL. Tumor cells expressing PD-L1 and PD-L2 can escape the immune surveillance of mature cytotoxic T cells *via* the PD-1 pathway, which is one of the most critical mechanisms that leads to cHL. 9p24 Genomic amplification of this.1, which may cause overexpression of PD-1, PD-L1, and PD-L2 in malignant HRS cells, is prevalent in this disease (38). PD-L1 blockers can restore immunoactivity in the tumor microenvironment (TME) and suppress the viability of HRS cells that express PD-1, PD-L1, and PD-L2. Nivolumab and pembrolizumab, which are both fully human IgG4 monoclonal PD-1 antibodies, were approved by the FDA in 2016 and 2017, respectively, as treatment options for patients with r/r cHL. The safety and efficacy of these two agents are not yet weighted, but healthcare insurance data suggest that the pembrolizumab cohort had a lower hospitalization rate than the nivolumab cohort (39). To date, finding novel and highly effective anti-PD-1 agents remains a major challenge worldwide. In China, several anti-PD-1 agents have been approved by the National Medical Products Administration for use in the treatment of r/r cHL from 2018 to 2022, including sintilimab (40, 41), tislelizumab (42, 43), camrelizumab (44–50), penpulimab (51), and zimberelimab (52). A summary of these agents is provided in **Table 2**.

**Table 2:** Anti-PD-1 antibodies approved by the National Medical Products Administration in China for r/r cHL.

Anti-PD-1 agent	Approved year	Patients	No. of patients	CRR (%)	ORR (%)	Severe toxicity (%)	NCT number
Sintilimab	2018	Relapsed or refractory after two or more lines of therapy	96	34	80·4	18	NCT03114683
Tislelizumab	2019	Failed or were ineligible for ASCT	70	67.1	87.1	31.4	NCT03209973
Camrelizumab	2019	Had prior ASCT or at least two lines of prior chemotherapy	75	28.0	76.0	26.7	NCT03155425
Enpulimab	2021	Had prior ASCT or at least two lines of prior chemotherapy	94	47.1	89.4	4.3	NCT03722147
Zimberelimab	2022	Relapsed or refractory after two or more lines of therapy	85	32.9	90.6	22.4	NCT03655483

The side effects of ICIs are non-specific, including infection, inflammation, pyrexia, digestive symptoms, etc. Notably, the side effect of nivolumab is statistically higher than that of pembrolizumab, and neutropenia is the most common side effect of nivolumab (53).

#### 3.2.1 Nivolumab

Nivolumab was the first ICI approved by the FDA to treat patients with r/r cHL. CheckMate 205 showed a durable response to nivolumab monotherapy in r/r cHL in 2018 (54), and the latest real-life experience in Turkey is consistent with this result in heavily pretreated r/r cHL (55). Analysis of cohort D of CheckMate 205 suggested that nivolumab combined with the AVD regimen (N-AVD) significantly increased ORR and CRR. The ORR and CRR at the end of nivolumab monotherapy were 69% and 18%, respectively, whereas, after two combination cycles of N-AVD, the figures increased to 90% and 51%, respectively (53). The randomized phase II NIVAHL trial explored the efficacy of N-AVD in an earlier stage of cHL as first-line therapy, which also resulted in an attractively high CR (56).

Nivolumab combined with BV has shown great outcomes as a second-line regimen, reporting an ORR of 82% and CR of 61% in r/r cHL (57). Another phase I study explored BV combined with nivolumab, nivolumab, and ipilimumab and found a similar ORR of 76% and CRR of 57% in the BV-nivolumab group and a high ORR of 82% and CRR of 73% in the triplet group (58). The next phase II study will compare these two modalities as a bridge to HCST (58). A comparison of the efficacy of nivolumab monotherapy and nivolumab in combination with ICE (NICE) multiagent therapy was conducted in a phase II trial (NCT03016871). Of the 42 evaluable patients, 34 received monotherapy, and nine received NICE therapy. The ORR and CRR in the NICE group were noticeably higher than those in the single-agent group (ORR 81% versus 93%; CRR 71% versus 91%) (59), although the number of samples was limited.

The ORR of anti-PD-1 agents was initially high. However, long-term treatment with nivolumab may decrease the drug efficacy and lead to chemoresistance. Nivolumab as salvage treatment without ASCT consolidation displayed a markedly higher relapse rate than those with ASCT consolidation (62.2% versus 0%), based on a retrospective analysis (60).

#### 3.2.2 Pembrolizumab

Pembrolizumab is another commonly used anti-PD-1 antibody approved by the FDA that has shown great PFS improvement (61–63). The phase III clinical trial of the KEYNOTE-204 study reported pembrolizumab as a preferred agent compared to BV in treating r/r cHL individuals who were ineligible for ASCT or had relapsed after ASCT. The median PFS reached 13.2 months for pembrolizumab versus 8.3 months for BV (*p* = 0.0027), although the ORR and CR did not show statistical significance ([65.6% (57.4–73.1) versus 54.2% (46.0–62.3) and 25% versus 24%, respectively) (64). Furthermore, pembrolizumab can improve health-related quality of life compared to BV in patients with r/r cHL (65). The duration of pembrolizumab response was observed when BV monotherapy was ineffective in the KEYNOTE-013 study (NCT01953692), and it was found that some patients may benefit from long-term pembrolizumab treatment since the duration of response was not reached in a 4-year follow-up (66).

Despite the remarkable efficacy of pembrolizumab as monotherapy, combinations of pembrolizumab and other agents are more widely suggested. A phase II study reported that pembrolizumab followed by AVD as first-line therapy was effective and well-tolerated in untreated patients with advanced stage cHL. Similarly, for r/r cHL patients, pembrolizumab also showed some favorable outcomes (67). A retrospective analysis reported a pembrolizumab-BV regimen in 10 patients with multirefractory cHL, and the final CR reached 80%. Seven out of 10 proceeded to ASCT directly, although the number of patients was limited, and further studies on two-drug therapy are of great significance (68). GVD, another commonly used salvage regimen of r/r cHL with a CRR of less than 50% (69), combined with pembrolizumab (pembro-GVD), was studied in a phase II clinical trial (NCT03618550) to evaluate its efficacy and safety. After two to four cycles of the pembro-GVD regimen, 95% of the patients achieved CR. Moreover, 36 out of 38 patients underwent HDT-ASCT, and no relapses were observed during a median follow-up of 13.5 months (70). AFM13, a CD30/CD16A-bispecific antibody that can stimulate innate immune cells, combined with pembrolizumab, has been reported to display good tolerance and an ORR of 88% in patients with r/r cHL (71); however, further investigation is needed.

#### 3.2.3 Sintilimab

Sintilimab is a highly selective PD-1 blocker that revealed an ORR of 80.4% in heavily pretreated cHL patients from 18 hospitals in China in a single-arm phase II trial (NCT03114683) (41). A new case was reported in which one cHL-HIV patient, who failed ABVD and GDP chemotherapeutic regimens, showed CR after receiving sintilimab for nine cycles, with acceptable adverse events (40).

#### 3.2.4 Tislelizumab

Tislelizumab is a humanized IgG4 monoclonal antibody that binds PD-1 with high affinity. A phase II trial in China evaluated the efficacy and safety of r/r cHL in patients who failed or were ineligible for ASCT, reporting an ORR of 87.1% and CR of 62.9% after a median of 9.8 months of follow-up (43). The latest update revealed that the PFS and OS rates were 40.8% and 84.8%, respectively, after a 3-year follow-up.

#### 3.2.5 Camrelizumab

Camrelizumab is an anti-PD-1 agent that has shown good efficacy against various advanced malignancies (44). It achieved a high rate of objective response (76.0%; 95% CI, 64.7–85.1) in cHL patients failing or ineligible for ASCT (45), and the median PFS was 22.5 months and the 36-month OS was 82.7%, according to the latest updates (46). However, some patients still have progressive disease or relapse after receiving anti-PD-1 agents, probably due to the overexpression of PD-1 in T cells in the microenvironment or upregulation of PD-L1 in HRS cells (47). Decitabine is a DNA methyltransferase inhibitor with the potential to reduce camrelizumab resistance (50). Therapy with camrelizumab plus decitabine markedly improved the CRR (71%) in patients with r/r cHL compared with camrelizumab monotherapy (32%) (48), and after a median follow-up of 34.5 months, the complete remission rate reached 79% with dual therapy (49). Further study of this combined therapy was conducted in two phase II trials (NCT02961101 and NCT03250962) in r/r cHL patients with PD-1 resistance, and the ORRs in two separate cohorts were 52% and 68%, respectively, with a longer PFS compared to PD-1 monotherapy (50). This suggests a high synergistic antitumor activity and long-term benefit of the camrelizumab plus decitabine combination. However, grade 3–4 adverse events of leukocytopenia occur more frequently with this combined therapy than with camrelizumab monotherapy.

## 4 Novel therapeutic measures to treat r/r cHL

Clinical Trials of potential effective novel agents in the treatment of r/r cHL under recruiting are summarized in **Table 3**. Although conventional therapeutic regimens, BV, and ICIs have greatly improved the prognosis, heavily chemoresistant patients still have a limited choice of effective drugs. Moreover, the high rate of adverse events among the aforementioned regimens remains a major obstacle, especially in adolescents and older patients. Therefore, novel agents that prolong PFS and OS and reduce adverse events are of great significance.

**Table 3:** Clinical trials of novel agents in the treatment of r/r cHL under recruiting.

Agents	Function	Intervention	Phase	NCT number
AZD7789	Anti-PD-1 and anti-TIM-3 antibody	Drug: AZD7789	Phase 1/2	NCT05216835
THOR-707	Non-α-selective IL-2	Drug: THOR-707 Drug: pembrolizumab	Phase 2	NCT05179603
Azacitidine	Cytosine nucleoside analogs	Biological: nivolumab Drug: oral azacitidine	Phase 1	NCT05162976
Ipilimumab	Anti-CTLA-4 monoclonal antibody	Drug: brentuximab vedotin Biological: ipilimumab Biological: nivolumab	Phase 1/2	NCT01896999
AZD4573	Cyclin-dependent kinase 9 inhibitor	Drug: AZD4573	Phase 2	NCT05140382
Ruxolitinib	JAK1/2 inhibitor	Drug: ruxolitinib Drug: nivolumab	Phase 1/2	NCT03681561
Decitabine	2-Deoxycytidine analogs	Drug: camrelizumab and decitabine	Phase 2/3	NCT04510610
Chidamide	Histone deacetylase inhibitor	Drug: chidamide Drug: camrelizumab Drug: decitabine	Phase 2	NCT04233294
TQB2450	Anti-PD-1 antibody	Drug: TQB2450	Phase 2	NCT03800706
CD30.CAR-EBVST cells	CD30.CAR in EBV-specific T cells	Biological: CD30.CAR-EBVST cells	Phase 1	NCT04288726
HSP-CAR30	Anti-CD30 CAR-T cells	Biological: HSP-CAR30	Phase 1/2	NCT04288726
AFM13	Anti-CD30/CD16A monoclonal antibody	Biological: AFM13 Drug: cyclophosphamide Drug: fludarabine Drug: fludarabine phosphate Biological: genetically engineered lymphocyte therapy	Phase 1	NCT04074746

### 4.1 Radioimmunotherapy

Since BV and ICIs are increasingly used in either the first-line or second-line treatment of cHL, the efficacy of these drugs may not be as effective as post-HCST maintenance therapy. Radioimmunotherapy is a novel targeted approach that may reduce the risk of relapse after HSCT in patients with radiosensitivity. 90Y-basiliximab/DOTA is a specially designed agent that conjugates basiliximab (anti-CD25 antibody) and 1,4,7,10-tetraazacyclododecane tetraacetic acid (DOTA) and radiolabels 90Y for therapeutic dosing. 90Y-basiliximab/DOTA was administered in combination with the BEAM regimen (carmustine, etoposide, cytarabine, and melphalan) as maintenance therapy, and the estimated 5-year PFS and OS rates were 68% and 95%, respectively (72). Further evaluation of this approach is ongoing in phase II trials.

### 4.2 Novel ADCs

ADC is a group of agents that connect small molecule bioactive drugs to monoclonal antibodies; hence, the antibodies can transport bioactive drugs to target cells directly. Because of the huge success of BV as an ADC in the treatment of cHL, novel ADCs against other key receptors are being investigated in both preclinical and clinical studies (73).

CD25 is a receptor of interleukin-2 (IL-2) that is abundantly distributed on the surface of both hematological tumor cells and Treg cells. Camidanlumab tesirine is a novel agent that conjugates anti-CD25 antibody to a pyrrolobenzodiazepine dimer and causes cell death by damaging the DNA structure. A phase I study assessed the efficacy of camidanlumab tesirine in patients with r/r cHL who had no available therapies. The median duration of response was 7.2 months, and the ORR was 71% (74, 75). A later phase II study demonstrated that 18 (38.3%) and 20 (42.6%) of 47 heavily treated patients attained CR and PR, respectively (76), showing that camidanlumab tesirine is a promising ADC agent next to BV.

CD123 is an alpha subunit of the interleukin 3 receptor (IL3RA) that is enriched in acute myeloid leukemia and cHL cells. IL3RA-directed ADC, such as BAY-943, seems to be effective in suppressing cHL development. BAY-943 has demonstrated noticeable antiproliferative efficacy in HL cell lines and xenograft models (77), and further clinical trials of BAY-943 are warranted.

### 4.3 Anti-CD30 CAR-T-cell therapy

CAR-T-cell immunotherapy has been increasingly studied in a wide range of cancers. Engineered T cells express CARs on the cell surface so that they can recognize and eliminate cells expressing specific target antigens. CAR-T therapy targeting the CD30 antigen (CD30.CAR-T) can be effective in hematological malignancies, including cHL (78). Adoptive transfer of CD30.CAR-T preceded by fludarabine-based lymphodepletion chemotherapy was performed in 41 heavily pretreated patients with r/r cHL who developed chemoresistance to the aforementioned agents, including BV and IPIs, with an ORR of 72%, CR of 59%, 1-year PFS of 36%, and OS of 94% (11). Although individuals that achieve CR after CD30.CAR-T could experience relapse again, possibly due to insufficient persistence of CAR-Ts, the combination of CD30.CAR-Ts and ICIs may improve their PFS, and further clinical trials are needed (11).

### 4.4 Potential effective chemotherapeutic agents and targeted agents

New combination regimens of standard chemotherapeutic agents have also been tested in an attempt to expand the range of options and achieve a higher prognosis. For example, gemcitabine and bendamustine are used in different conventional regimens, while their concurrent combination was first reported in a phase I/II study as late-line therapy for heavily pretreated patients, showing a well-tolerated and effective result (ORR 69%; CRR 46%) but a potentially high pulmonary toxicity risk (79).

Novel chemotherapeutic agents are also being researched in both preclinical and clinical studies. Lenalidomide is an immunomodulatory drug used to treat myelodysplastic syndrome, and a low dose displays some activity in patients with r/r cHL who fail or are ineligible for ASCT, with 64% of the patients remaining stable and 11% achieving a PR. However, it is not suggested for post-ASCT treatment (80, 81). A phase I/II clinical trial combined lenalidomide with the mTOR inhibitor temsirolimus in patients with r/r cHL and reported an ORR of 80% and a CR of 35% (82), which is encouraging. Trabectedin is a tetrahydroisoquinoline alkaloid with antitumor activity targeting both TME and HRS cells in cHL. Preclinical studies have demonstrated that trabectedin induces DNA skeleton cleavage and cancer cell necrosis by binding to the DNA structure and blocking the transcription level of stress-induced proteins. It also reduced HRS cell secretion of important factors and reduced immunosuppressive immunocytes in the TME, indicating that trabectedin can be a rational candidate in patients with r/r cHL (83). Furthermore, the combination of trabectedin and the CCR5 antagonist maraviroc enhanced DNA damage and antitumor activity (84).

## 5 Conclusion

cHL is a generally curable disease that occurs mainly in those aged 15–30 and over 55 years (3). However, a small group of patients are refractory to treatment or suffer a relapse after receiving primary systemic therapy and HDT-ASCT. The prognosis for these patients is notably poor. In this review, we briefly summarized the current treatment principles and conventional regimens and provided an update on the latest clinical trials of leading cHL agents (BV and ICIs) and novel potential drugs that may be used in the near future. In addition, prognosis evaluation plays a vital role in directing the next treatment strategy and analyzing the risk of relapse or disease progression. The International Prognostic Score (IPS) is composed of seven potential risk factors [albumin < 4 g/dl, hemoglobin < 10.5 g/dl, male, age ≥ 45 years, stage IV disease, leukocytosis, and lymphocytopenia (5)] and is intensively applied for risk classification. PET/CT is currently the most effective method to assess treatment efficacy and predict individual prognosis. The Deauville criteria is the most commonly used scale to evaluate the degree of lymphoma remission. However, when comparing different clinical trials, ORR and CRR may be based on different criteria, among which the Lugano 2014 classification and 2007 revised response criteria are frequently used. Although the guidelines for adult patients with cHL are mature, the treatment of certain groups of patients (including pediatric, older, and HIV-related individuals) (85) remains controversial. Further studies should focus on these population groups.

## Author Contributions

YZ, TW, and WW designed and analyzed the manuscript and were responsible for the funding acquisition and manuscript writing—review and editing. ZX, LM, ZL, and JZ supervised the study and drafted the manuscript. All authors reviewed and approved the manuscript.

## Funding

This work was financially supported by the Support Program for Science and Technology Department of Sichuan Province (2021YFS0228, 2021YFS0230) and the 1·3·5 Project for Disciplines of Excellence, West China Hospital, Sichuan University (ZYJC21033).

## Conflict of Interest

The authors declare that the research was conducted in the absence of any commercial or financial relationships that could be construed as a potential conflict of interest.

## Publisher’s Note

All claims expressed in this article are solely those of the authors and do not necessarily represent those of their affiliated organizations, or those of the publisher, the editors and the reviewers. Any product that may be evaluated in this article, or claim that may be made by its manufacturer, is not guaranteed or endorsed by the publisher.
